# Changes in skeletal muscle gene expression following clenbuterol administration

**DOI:** 10.1186/1471-2164-7-320

**Published:** 2006-12-20

**Authors:** Diane M Spurlock, Tara G McDaneld, Lauren M McIntyre

**Affiliations:** 1 Department of Animal Sciences, Iowa State University, Ames, IA, USA; 2 Department of Molecular Genetics and Microbiology, University of Florida, Gainesville, FL, USA

## Abstract

**Background:**

Beta-adrenergic receptor agonists (BA) induce skeletal muscle hypertrophy, yet specific mechanisms that lead to this effect are not well understood. The objective of this research was to identify novel genes and physiological pathways that potentially facilitate BA induced skeletal muscle growth. The Affymetrix platform was utilized to identify gene expression changes in mouse skeletal muscle 24 hours and 10 days after administration of the BA clenbuterol.

**Results:**

Administration of clenbuterol stimulated anabolic activity, as indicated by decreased blood urea nitrogen (BUN; *P *< 0.01) and increased body weight gain (*P *< 0.05) 24 hours or 10 days, respectively, after initiation of clenbuterol treatment. A total of 22,605 probesets were evaluated with 52 probesets defined as differentially expressed based on a false discovery rate of 10%. Differential mRNA abundance of four of these genes was validated in an independent experiment by quantitative PCR. Functional characterization of differentially expressed genes revealed several categories that participate in biological processes important to skeletal muscle growth, including regulators of transcription and translation, mediators of cell-signalling pathways, and genes involved in polyamine metabolism.

**Conclusion:**

Global evaluation of gene expression after administration of clenbuterol identified changes in gene expression and overrepresented functional categories of genes that may regulate BA-induced muscle hypertrophy. Changes in mRNA abundance of multiple genes associated with myogenic differentiation may indicate an important effect of BA on proliferation, differentiation, and/or recruitment of satellite cells into muscle fibers to promote muscle hypertrophy. Increased mRNA abundance of genes involved in the initiation of translation suggests that increased levels of protein synthesis often associated with BA administration may result from a general up-regulation of translational initiators. Additionally, numerous other genes and physiological pathways were identified that will be important targets for further investigations of the hypertrophic effect of BA on skeletal muscle.

## Background

Anabolic effects of β-adrenergic receptor agonists (BA) have been widely studied for potential applications in the prevention of muscle atrophy [1,2] and improvement of the efficiency of muscle growth in production livestock [3-5]. Clenbuterol is a β2-adrenergic receptor agonist that has been shown to have a significant effect on muscle metabolism in a variety of muscle atrophy models, including hind-limb suspension atrophy [6,7], starvation induced atrophy [8], and denervation induced atrophy [9,10]. Additionally, clenbuterol is known to induce a significant repartitioning effect by increasing the growth of skeletal muscle at the expense of fat tissues in most livestock species [3,5]. Although it is known that clenbuterol initiates these effects via activation of the β2-adrenergic receptor [3,5], the downstream mechanisms by which activation of these receptors results in increased muscle growth or decreased muscle atrophy are not clear. To date, the expression and activity of specific genes have been investigated in a variety of models in order to implicate specific pathways with the skeletal muscle response to BA. For example, increased abundance of myofibrillar and structural proteins has been demonstrated and appears to result from increases in both transcription and translation of these genes [11-13]. Additionally, endogenous proteinases including genes of the ubiquitin-proteasome pathway and calcium-dependent proteolytic enzymes have been reported to mediate protein turnover in skeletal muscle after administration of BA [14]. Finally, changes in skeletal muscle expression of IGF1 and IGF2 mRNA have been observed shortly after the administration of clenbuterol to rodents [15], suggesting the regulation of these growth factors may be important in the initial response of skeletal muscle to BA.

Although investigations of candidate genes have been informative in terms of implicating individual pathways in the skeletal muscle response to BA, they have not provided a global view of changes occurring in the tissue, and have been limited to the investigation of genes with known functions. Thus, the objective of the current research was to define changes in the global gene expression profile of skeletal muscle in response to administration of the BA clenbuterol. Two time points relative to clenbuterol administration were investigated in order to compare gene expression profiles following short- (24 hour; 24 h) and long- (10 day; 10 D) term clenbuterol administration in mice. The Affymetrix platform was chosen for the analysis of gene expression in order to investigate the most comprehensive collection of genes available.

## Results

### Clenbuterol stimulated an anabolic response

**Figure 1:**
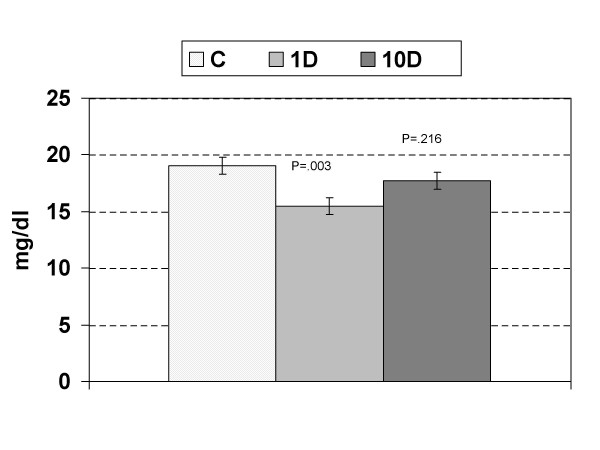
**Blood urea nitrogen (BUN) levels verify ananabolic response**. Blood urea nitrogen levels of mice following ten days of injections, including 24 hours (24 h) or 10 (10 D) days of clenbuterol administration, or a vehicle control (C). The overall effect of treatment on BUN was significant (*P *< 0.01), and significance values from contrasts comparing each clenbuterol treatment to the control group are shown.

**Figure 2:**
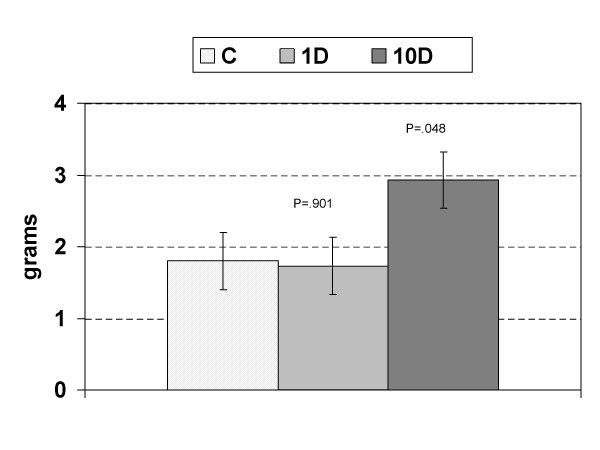
**Body weight gain measurements verify an anabolicresponse**. Body weight gain of mice following ten days of injections, including 24 hours (24 h) or 10 (10 D) days of clenbuterol administration, or a vehicle control (C). The overall effect of treatment on body weight gain showed a trend for significance (*P *= 0.06), and significance values from contrasts comparing each clenbuterol treatment to the control group are shown.

A significant effect of clenbuterol on blood urea nitrogen (BUN) was observed due to decreased BUN in 24 h compared to control (C) treatment groups (*P *< 0.01; Figure 1). Although average BUN of 10 D treated mice was less than that of C mice, this difference was not statistically significant (*P *> 0.05). Body weight gain tended to differ among the three groups of mice (*P *= 0.06), with a significant increase in body weight gain observed following 10 D clenbuterol administration compared to the C group (*P *< 0.05; Figure 2).

### Clenbuterol stimulated changes in mRNA abundance

A total of 22,605 probesets were deemed detected across the MOE430A and MOE430B chips and were included in all subsequent analyses. All data were deposited in the GEO data base (Accession number GSE4490). A total of 137, 56 and 4 probesets were differentially expressed based on false discovery rates (FDR) of 20, 10, and 5%, respectively, which correspond to *P*-values less than 0.0012, 0.00025, and 0.0000082, respectively. We considered genes that were significant at the 10% FDR threshold significantly differentially expressed. While individual nominal significance levels are often set at 5%, when multiple tests are performed the impact of both the type I (false positive) and type II (false negative) error rate should be carefully considered. The FDR provides a way of controlling the expected number of false positives in the list of tests rejected [16,17]. With 56 rejections a 10% FDR says that the expectation is less than six false positives. In this case, the substantial increase in the number of genes identified is much larger than the anticipated increase in type I error and so we opted to place more weight on reducing the type II error and increasing power. Among these 56 probesets, four displayed evidence for non-normal distributions of residuals. In order to be conservative we do not consider these further, leaving 52 probesets deemed differentially expressed (Table 1). The mRNA abundance of 63% of these probesets differed at both 24 h and 10 D, with 24 transcripts having consistently more and 8 consistently less mRNA relative to C at both time points. One gene, A530047J11Rik, changed in opposite directions relative to C in the 24 h and 10 D treatment groups. A total of 20 probesets showed altered mRNA abundance in the 24 h but not 10 D treatment, with approximately equal numbers increasing (11) and decreasing (8) relative to the C group.

**Figure 3:**
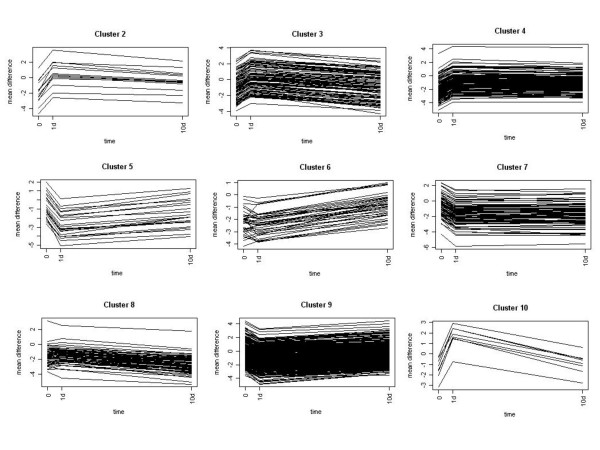
**Clustering of all expressed genes according to themethod of orthogonal polynomials**. Ten clusters representing different patterns of mean expression changes for response to clenbuterol. The number of genes in each cluster is given in Table 2. Cluster 1 is not shown because minimal change in gene expression was apparent. Specific genes included in each cluster are given in Additional File 1.

A recent paper from Yi and Xu 2006 [18] describes a method for grouping or clustering genes based upon a time series that places genes with similar expression patterns, regardless of statistical significance, into clusters. We applied this technique to our data and found that the 22,605 detected genes were grouped into a total of 10 clusters describing gene expression profiles over time (Figure 3). The number of probesets included in each cluster ranged from eight to 21,738 (Table 3), with the largest group representing genes with a 'flat' profile, or no evidence of changes in gene expression. However, this method identified 867 genes whose mean gene expression changed over time. Expression changes represented by each cluster are shown in Figure 3, and the cluster to which each probeset belongs is given in Supplementary Table 1.

The discrepancy between the number of genes whose mean behaviour appears different, and the number which are statistically significant after correction for multiple tests, may be indicative of weaker signals in these data than in many array experiments where the treatment conditions produce a dramatic direct response. If clenbuterol indirectly affects a gene, by perhaps targeting an upstream regulator, the magnitude of the effect seen in the downstream targets would be lower. In order to include genes potentially indirectly regulated by clenbuterol administration in our exploratory analysis of the functional groups underlying response to clenbuterol, a nominal threshold of *P *< 0.01 was used. This group of 575 genes was examined further to help identify biological processes potentially influenced by clenbuterol treatment. A total of 309 of these genes were categorized into 24 Gene Ontology biological process categories (Level 2) with two or more members, while 242 genes remained unclassified (Table 4). Categories with the greatest number of genes are consistent with the model of skeletal muscle hypertrophy. Analysis results for all genes, including the overall and contrast *P*-values, cluster, and mean expression for each treatment (for log transformed and raw data) are provided in Supplementary Table 1.

### Clenbuterol stimulated changes in families and categories of genes

Five hundred and seventy five genes (*P *< 0.01) were further evaluated using EASE score [19] to identify categories of over-represented genes, based on the Gene Ontology biological process annotation. Briefly, the EASE score compares the proportion of differentially expressed genes found within a category to the proportion of genes in that category detected from the GeneChip. The EASE score test statistic accounts for issues related to multiple testing across many biological process categories in a semi-conservative manner [19]. A total of 19 biological process categories were over-represented in our set of genes of interest, based on an EASE score < 0.05. After accounting for redundant categories, the differentially expressed genes were classified into 10 over-represented groups (Table 5). Categories of particular interest to the model of clenbuterol stimulated muscle hypertrophy include: intracellular signaling cascade, amino acid and derivative metabolism, translation, and transcription from the Pol II promoter. Specific genes from these categories are described in detail in the Discussion and highlighted in Table 1 and Table 2. The list of genes of interest was also searched for members that had previously been associated with BA induced muscle hypertrophy, including structural genes, IGF1 and its receptors and binding proteins, and genes involved in ubiquitination and proteasomal degradation (Table 2).

### Validation of differential gene expression

Differential expression of four genes was validated in an independent experiment using a different set of mice from the microarray experiment. Genes were selected from the list of statistically significantly differentially expressed genes to represent different biological processes important to muscle physiology, including genes that interact with structural proteins (Arpc3 and vinculin), a regulator of transcription (Hod), and a growth factor implicated in multiple facets of muscle growth and differentiation (IGF1). In concordance with the microarray data, mRNA abundance of each of these genes increased 24 h after administration of clenbuterol treatment relative to C animals (*P *< 0.05), while GAPDH represented a housekeeping gene whose mRNA abundance did not differ between treatments (Table 6). Thus, these data provide strong biological validation of results from the GeneChip experiment.

## Discussion

Although it has been known for decades that BA stimulate muscle hypertrophy [5], the data described herein are the first to provide an overview of global changes in gene expression associated with this biological model. These results also contribute to a growing body of literature describing gene expression changes associated with various models of skeletal muscle hypertrophy and atrophy [20-22]. Together, these data facilitate the opportunity to define genes across multiple models of muscle physiology, as well as to identify genes specific to the effects of BA. One unique aspect of our experiment is the investigation of gene expression at two time points relative to the administration of clenbuterol. Previous studies have focused on altered gene and/or protein expression after significant changes in muscle mass have occurred [12,13,23]. However, results of our study indicate that changes in gene expression are equally abundant at an early time point relative to the initiation of clenbuterol administration. These initial changes may represent important alterations of physiological pathways that culminate in altered protein turnover and/or recruitment of satellite cells to support muscle growth.

Our analysis showed that four of the 56 differentially expressed genes (10% FDR) met an even more stringent 5% FDR threshold: cyclin dependent kinase inhibitor 1A (p21) (Cdkn1a), TSC22 domain family, member 1 (Tsc22d1), growth hormone receptor (GHR), and tubulointerstitial nephritis antigen-like (Tinagl). Two of these genes, Cdkn1a and GHR, are of particular interest to this experimental model because of their well-established roles in muscle growth and development [24-28]. Cdkn1a inhibits cyclin dependent kinase 2 activity, contributing to the irreversible withdrawal from the cell cycle and terminal differentiation of myocytes [24]. Although the presence of Cdkn1a is not required for normal development of mice [25], the absence of this gene product prevents normal regeneration of muscle through satellite cells [26]. Therefore, the significant upregulation of Cdkn1a mRNA abundance following both short and long-term clenbuterol administration in our experiment may suggest an increased potential for terminal differentiation and recruitment of myogenic precursor cells in support of muscle hypertrophy. GHR is the transmembrane receptor for growth hormone (GH), a hormone essential for normal growth [27]. Although GH effects are largely mediated through the stimulation of synthesis and secretion of insulin-like growth factor 1 (IGF1), it has been demonstrated that GH has important roles in the postnatal regulation of skeletal muscle growth that are independent of IGF1 [28]. This work demonstrates GH signalling from GHR influences muscle hypertrophy by facilitating the fusion of myoblasts with myotubes [28]. Our data reveal a significant down-regulation of GHR in skeletal muscle following one and ten days of clenbuterol administration. This change in GHR expression is consistent with previous reports of decreased GHR mRNA in a compensatory overload model [29], and fiber-type specific increased GHR mRNA in a hindlimb suspension model of muscle atrophy [30]. Together, these reports clearly implicate GHR as a potential regulator of skeletal muscle growth and atrophy across multiple experimental models.

The EASE classification of potential genes of interest also identified overrepresented functional categories known to be important in muscle physiology and growth. Closer evaluation of genes within these categories reveals multiple pathways that potentially contribute to BA induced muscle hypertrophy. Each of the genes discussed below is included in Table 1 or 2, which include the level of significance, fold and direction of change for each gene. Additionally, all genes grouped in the categories of interest described below are identified in Supplementary Table 1.

### Amino acid and derivative metabolism

The group of 16 genes categorized as being involved in 'amino acid and derivative metabolism' includes three genes critical to polyamine metabolism (S-adenosylmethionine decarboxylase 1 [Amd1; *P *= 0.00015], ornithine decarboxylase, structural 1 [Odc1; *P *= 0.00037], and spermine oxidase [Smox; *P *= 0.00745]). Polyamines, including spermine, spermidine, and putrescine, are polycationic compounds found in both prokaryotic and eukaryotic cells that are known to be crucial to growth and proliferation of mammalian cells [31-34]. The three genes differentially expressed in our experiment represent critical steps in the biosynthesis of polyamines. Polyamines have previously been associated with cardiac hypertrophy stimulated by BA [35-37]. For example, the Odc1 inhibitor DFMO successfully blocked cardiac hypertrophy normally associated with clenbuterol [35,36], and overexpression of Odc1 in a transgenic model resulted in a significant increase in BA stimulated cardiac hypertrophy relative to non-transgenic mice [37]. These experiments demonstrate a clear interaction between polyamines and beta adrenergic receptor stimulated cardiac hypertrophy. However, the relationship between polyamine metabolism and beta adrenergic receptors remains to be defined for skeletal muscle. Our data suggest that clenbuterol administration increases mRNA abundance of Odc1 while decreasing the abundance of Amd1 and Smox. This differential regulation is difficult to interpret since each of these genes participate in the synthesis of polyamines. However, increased Odc1 expression is consistent with cardiac models that demonstrate an interaction between Odc1 activity and BA. It is also of interest to note that mRNA abundance was altered for two genes that function in the transport of Arginine (solute carrier family 7 [cationic amino acid transporter, y+ system], members 2 and 5 [Slc7a2; *P *= 0.00051 and Slc7a5; *P *= 0.00012]), the precursor of ornithine. However, mRNA abundance for these transporters changed in opposite directions following clenbuterol administration. The identification of differential expression of multiple genes involved in polyamine metabolism, combined with previous data linking polyamines to BA induced cardiac hypertrophy define polyamine metabolism as a critical target for further investigation of BA induced skeletal muscle growth.

### Translation

This gene ontology category was represented by 13 genes, including four eukaryotic translation initiation factors. Eukaryotic initiation factor 2 (Eif2) is involved in the first step of translation through the formation of a ternary complex between the initiator tRNA and GTP [38,39]. This complex then binds to the 40S ribosomal subunit to initiate translation. Following start codon recognition, GTP is hydrolyzed and the resulting GDP-Eif2 complex is released. A new cycle of initiation of translation requires Eif2b to catalyze the exchange of Eif2-bound GDP for GTP, which is an important step in the regulation of translation initiation. Because Eif2b is present in low amounts, it is an important factor controlling the global rate of protein synthesis [38,39]. Our data show that mRNA abundance of two subunits of Eif2b, Eif2b4 (*P *= 0.00325) and Eif2b2 (*P *= 0.00690), are upregulated in response to clenbuterol administration. Increased abundance of these subunits may contribute to increased activity of Eif2b and the global upregulation of protein synthesis in skeletal muscle previously associated with clenbuterol administration [40,41]. An association between Eif2b and adrenergic receptors has also been described as Eif2b1 directly interacts with the beta-2 adrenergic receptor [42]. Overexpression of Eif2b1 in 293 cells caused a small but significant increase in beta adrenergic receptor signalling activity. Although expression of Eif2b1 was not altered in our experiment, it is not known if the effect of Eif2b1 on beta receptor activity depends on association with other proteins, such as Eif2b2 and Eif2b4 that may be present in limiting quantities. Therefore, the observed upregulation of Eif2b2 and Eif2b4 in our study may represent a mechanism by which clenbuterol administration enhances signalling activity from beta adrenergic receptors. In addition to these Eif2b subunits, elongation initiation factor 5 (Eif5; *P *= 0.00679) and elongation initiation factor 4g3 (Eif4g3; *P *= 0.00971) also had increased mRNA abundance 24 hours after clenbuterol administration. Together, these results are consistent with a general upregulation of translational machinery occurring shortly after the administration of clenbuterol.

### Transcription from Pol II promoter

A total of 18 genes were found in this category, including four genes with known functions in muscle growth and development: homeobox only domain (Hod; *P *= 0.00012), YY1 transcription factor (Yy1; *P *= 0.00004), myocyte enhancer factor 2C (Mef2c; *P *= 0.00368), and myogenin (Myog; *P *= 0.00703). The YY1 transcription factor has been associated with myogenic differentiation [43]. In skeletal muscle, Yy1 inhibits transcription of the alpha actin gene, and down-regulation of Yy1 is necessary for alpha actin expression and myogenic differentiation to proceed [43]. Our data show a significant increase in Yy1 mRNA abundance following 24 hours and 10 days of clenbuterol administration, potentially contributing to decreased differentiation of myogenic cells. However, alterations in Yy1 protein abundance have been shown to occur through proteolytic regulation, independent of changes in mRNA [43]. Thus, future investigation of the regulation of Yy1 in response to BA stimulation will require careful consideration of changes at both the mRNA and protein level. Another transcription factor that was differentially expressed in our experiment was Hod, an unusual homeodomain protein that modulates cardiac growth and development [44,45]. Hod functions by interacting with serum response factor (SRF) to inhibit activation of SRF-dependent transcription [46]. Inactivation of Hod in transgenic mouse models confirm that an absence of Hod results in an imbalance between proliferation and differentiation of cardiomyocytes, culminating in impaired cardiac development [44,45]. In contrast, transgenic mice that overexpress Hod develop severe cardiac hypertrophy that is mediated by inhibition of SRF-dependent transcriptional activity. Our data show a significant increase in Hod mRNA 24 hours after clenbuterol administration. Although the effect of Hod on skeletal muscle growth and differentiation has not been described, its effect on cardiac hypertrophy makes it an intriguing candidate as a potential mediator of the growth promoting effects of clenbuterol. Additionally, the regulation of Hod by BA may have important implications regarding cardiac hypertrophy commonly observed in response to these compounds [45]. Myogenin and Mef2c are well characterized transcription factors known to be essential for myoblast differentiation [47,48]. Although significant alteration of satellite cell differentiation and recruitment into muscle fibers in response to BA has not been described, the observed increase in mRNA of transcription factors that contribute to muscle cell differentiation suggest the recruitment and differentiation of pre-myogenic cells may be involved in the physiological response of skeletal muscle to clenbuterol administration.

### Intracellular signalling cascade

Of the 34 genes of interest categorized as intracellular signalling molecules, four belong to the mitogen activated protein (MAP) kinase signalling pathway (growth arrest and DNA-damage-inducible 45 gamma [Gadd45g; *P *= 0.00015], mitogen activated protein kinase kinase 4 [Map2k4; *P *= 0.00492], mitogen activated protein kinase kinase 6 [Map2k6; *P *= 0.00552], and protein phosphatase 1 (formerly 2C)-like [Ppm11; *P *= 0.00689]). The MAP kinase signal transduction pathway is of particular interest to this skeletal muscle model because it is a key mediator of the cellular response to insulin-like growth factor 1 (IGF1), a known regulator of myogenic cell proliferation, differentiation, and protein turnover [49,50]. In our experiment, mRNA abundance of IGF1 increased 24 hours after clenbuterol administration (*P *= 0.00018), but was not different from C after 10 days. Inconsistent results describing the effect of BA on IGF1 have been reported, but these likely reflect differences in the timing and sampling (local IGF1 production versus circulating levels) across experiments. Additionally, IGF2 receptor (*P *= 0.00065) and IGF binding protein 5 (*P *= 0.00051) had decreased mRNA abundance at both 24 h and 10 D time points. Although our experiment was not designed to clarify the relationship between IGF1 and BA stimulated muscle growth, it does provide data suggesting an interaction between these pathways. One potential explanation is that cross-talk between these pathways facilitates complex regulation of the recruitment of myogenic precursor cells or protein turnover within muscle fibers.

The intracellular signalling cascade category also includes three members of the ankyrin repeat and SOCS-box (Asb) containing gene family: Asb10 (*P *= 0.00022), Asb14 (*P *= 0.00245) and Asb15 (*P *= 0.00001). Members of this gene family have been shown to act as an E3 ligase to target specific proteins for degradation through the ubiquitin-proteasome degradation pathway [51-53]. Although specific proteins targeted for degradation through interactions with Asb10, Asb14 and Asb15 have not been identified, we have previously reported that Asb15 mRNA is down-regulated in response to BA in other animal models [54]. Additionally, we have demonstrated that localized over-expression of Asb15 stimulates muscle hypertrophy *in vivo*, and that its over-expression causes a delay in differentiation and increase in protein synthesis in C2C12 cells [55]. The current experiment provides additional support for the hypothesis that Asb family members participate in the regulation of intracellular signalling via beta adrenergic receptors by showing that multiple members of the Asb family are coordinately regulated at the mRNA level. Although much remains to be learned about the Asb gene family and its role in targeting proteins for ubiquitination, the down-regulation of three members of the Asb family supports the idea of changes in the turnover of specific, targeted proteins in response to BA.

The ubiquitin-proteasomal pathway is known to be a major contributor to protein degradation in skeletal muscle, and decreases in components of the ubiquitin-proteasome pathway have been associated with decreased protein degradation leading to muscle hypertrophy [56]. Additionally, administration of clenbuterol has been reported to alter expression of ubiquitin-proteasome conjugates in mice following hindlimb suspension [14]. Although the EASE analysis of our data did not identify ubiquitin related genes to be significantly over-represented, three genes involved in the ubiquitin-proteasomal pathway, including ubiquitin protein ligase E3 component n-recognin 1 [Ubr1; *P *= 0.00165], seven in abstentia 2 [Siah2; *P *= 0.00460], and proteasome subunit, beta type 1 [Psmb1; *P *= 0.00066], were differentially expressed. It should also be noted that the Asb gene family members previously discussed were not associated with gene ontology terms related to the ubiquitin pathway at the time of analysis. The mRNA abundance of the three ubiquitin related genes was upregulated following clenbuterol administration in our experiment. This result was unexpected because an increase in components of the ubiquitin-proteasomal pathway is associated with increased protein degradation [57,58], while clenbuterol is known to decrease protein degradation [3]. One potential explanation for this finding is that skeletal muscle undergoes significant fiber-type transition in response to clenbuterol administration, and the ubiquitin-proteasome system has been implicated in this process [59]. Therefore, our results may reflect changes in skeletal muscle specifically associated with fiber-type transition, rather than a general increase in protein degradation.

### Structural proteins

Few examples of structural proteins typically associated with skeletal muscle hypertrophy were found in the list of differentially expressed genes, although myosin IB [Myo1b; *P *= 0.00578], actinin alpha 4 [Actn4; *P *= 0.00613], and myosin XVIIIa [Myo18a; *P *= 0.00377] had altered mRNA abundance associated with clenbuterol administration. Additionally, genes encoding proteins that interact with structural proteins were regulated. For example, actin related protein 2/3 complex subunit [Arpc3; *P *= 0.00005], and vinculin [Vcl; *P *= 0.00002] increased in mRNA abundance after administration of clenbuterol. Vinculin is an integrin-associated protein located at junctions between actin and the plasma membrane, while Arpc3 is a subunit of the Arp2/3 complex which functions in actin remodeling [60-62]. Vinculin is thought to facilitate actin organization by recruiting the Arp2/3 complex which binds to actin [60-62]. Both Arpc3 and Vcl had increased mRNA abundance following clenbuterol administration. Thus, it is reasonable to hypothesize that Arpc3 and Vcl are upregulated in the present study in order to support an increase in structural proteins associated with muscle hypertrophy.

## Conclusion

Global evaluation of gene expression after administration of clenbuterol identified changes in gene expression and overrepresented functional categories that may regulate BA-induced muscle hypertrophy. Changes in the mRNA abundance of multiple genes associated with myogenic differentiation may indicate an important effect of BA on the proliferation, differentiation, and/or recruitment of satellite cells into muscle fibers to promote muscle hypertrophy. Additionally, increased mRNA abundance of genes involved in the initiation of translation suggests that increased levels of protein synthesis often associated with BA administration may result from a general up-regulation of translational initiators, rather than a sustained up-regulation of gene expression at the transcriptional level. Finally, numerous other genes and physiological pathways were identified that will be important targets for further investigations of the hypertrophic effect of BA on skeletal muscle.

## Methods

### Animal management and tissue collection

A total of 36 C57BL/6J male mice (Jackson Labs, Bar Harbor, ME), 3 to 5 wk of age, were utilized in the experiment. Mice were housed three per cage and maintained at 25°C with a 12:12-h light-dark cycle and *ad libitum *access to water and feed (Rodent Laboratory Chow, Ralston Purina, St. Louis, MO) throughout the experiment. All animals were handled in accordance with the protocol approved by the Purdue Animal Care and Use Committee. Four cages (three mice per cage) were randomly assigned to one of three experimental treatments (n = 12 mice per treatment): vehicle control (C), 24 hour clenbuterol treatment (24 h), or 10 day clenbuterol treatment (10 D). Clenbuterol (Sigma, St. Louis, MO) was prepared by dissolving 2.5 mg in 1 mL of ethyl alcohol, diluting to 0.25 mg/ml with 1:1 PEG200:phosphate buffered saline, and sterilized by filtration. The vehicle control treatment was prepared in the same manner, except that no clenbuterol was added. Following a seven day acclimation period, all mice were administered daily intraperitoneal injections of control or clenbuterol treatments for ten days. The C group received the control treatment all ten days, the 24 h group received the control treatment for nine days and clenbuterol on day 10, and the 10 D group received clenbuterol for all ten days. The clenbuterol treatment was administered at a dosage of 1 mg/kg of body weight, and an equivalent volume of the control treatment was injected. All mice were euthanized by CO_2 _asphyxiation 24 hours following the tenth injection. The gracillus muscle was collected from each animal, immediately frozen in liquid nitrogen, and stored at -80°C pending RNA extraction. The gracillus muscle was selected for analysis since it contains a mixed fiber type of both Type I and Type II fibers.

Body weight change and blood urea nitrogen level (BUN) were measured as indicators of a physiological response to the clenbuterol treatment. Individual body weights were recorded daily at the time of treatment administration (approximately 10 am), and body weight gain (WG) was calculated as the difference in body weight between days ten and one. Truncal blood was collected at the time of tissue collection, immediately following euthanasia. Serum was isolated and stored at -20°C pending analysis. Blood urea nitrogen was measured via methods of Kerscher and Ziegenhorn [63]. WG and BUN were evaluated by analysis of variance using the GLM procedure of SAS [64] to test the main effect of clenbuterol treatment. Means of the 24 h and 10 D groups were compared to the C group using contrasts to determine if differences were significant.

### RNA isolation and hybridization

A total of 27 mice (9 per treatment) were selected in order to maximize the difference in BUN and WG between the 24 h and 10 D treatments relative to C. Total RNA was extracted from the gracillus muscle of each mouse using the Qiagen RNeasy Mini kit following the manufacturer's recommended protocol, including an additional step of protein kinase digestion for muscle tissue (Qiagen, Valencia, CA). Quality and quantity of individual RNA samples were examined using a 2100 Bioanalyzer (Agilent, Palo Alto, CA). Three pools of RNA representing each treatment were made. Mice were randomly assigned to pools within treatment (three mice per pool) such that each pool contained an equal quantity of RNA from each mouse, for a total of greater than 50 μg of RNA at a concentration of at least 2.5 μg/μl.

Messenger RNA, cRNA synthesis and labelling reactions were performed independently for each replicate following the recommendations of the Gene Chip^® ^Expression Analysis technical manual (Affymetrix, Santa Clara, CA). The MOE430A and B chips were hybridized to the fragmented cRNA, stained and washed according to the recommendations of the Gene Chip^® ^Expression Analysis technical manual (Affymetrix, Santa Clara, CA) in the Purdue University Genomics Core facility. Image data were quantified using GeneChip Analysis Suite/Microarray Suite 5.0 (MAS 5.0).

### Statistical analysis

If all replicates for a particular probeset were deemed 'absent', that probeset was removed from further consideration (n = 22,478) and the remaining probesets were analyzed (n = 22,605). Transcript levels were normalized to the chip median and log transformed. For each probeset, which represents the combined expression data from all relevant probe pairs on the chip, the generalized linear model *Y*_*ij *_= *μ *+ B_1_*T*_*i *_+ ε_ij _was fit. In each ANOVA, *Y*_*ij *_is a the log normalized transcript level for the i^th ^treatment and the j^th ^replicate, *μ *is the overall mean expression for the feature and *T*_*i *_represents the i^th ^treatment (C, 24 h, and 10 D). An F test of the effect of treatment for each probeset was conducted as the ratio of the mean squares for treatment over the mean squares for error, and the *P*-value for the test of the null hypothesis t_2 _= t_1 _= t_0 _(i.e., mean expression not different among the three treatments) was calculated. We examined the model for conformation to the assumption of normality of the residuals testing the null hypothesis that the residuals for each gene were normally distributed using the Shapiro-Wilkes Test. All analyses were performed in SAS (SAS Institute, Cary NC [64]). An FDR [17] level of 10% was used for declaring findings significant, and a stringent rate of 5% was also examined. A nominal threshold of *P *< 0.01 was defined to identify genes of potential interest for the EASE analysis. If the test of the null hypothesis of difference across treatments was rejected, and we had no evidence for departure from normality of the residuals, we declared the gene differentially expressed across treatments and qualitatively assessed additional contrasts comparing the effect of the treatments (C versus 24 h and C versus 10 D).

We used the orthogonal polynomials method recently described in Yi and Xu 2006 [18] to group the 22,605 probesets into clusters. Briefly, genes are clustered based on non-linear association between gene expression and time using orthogonal polynomials under the Gaussian mixture model. The whole data set is assumed to be a mixture of *K *clusters. The gene expression for individual probesets is the sum of a cluster mean (fixed effect), a normally distributed random effect that specifies deviation from the cluster mean, and a random measurement error. All genes are centered so that average expression is zero before clustering. In this way, clustering is based solely on the pattern of association of their expression with time and not the magnitude of gene expression. Bayesian Information Criterion (BIC) is used to choose the optimal number of the clusters.

### Functional characterization of differentially expressed genes

Gene Ontology biological process categories were defined for genes of interest using DAVID [65] with the Entrez Gene ID as the primary identifier. Over-represented functional categories were identified using the EASE score [16], as determined by the Expression Analysis Systematic Explorer (EASE) software [66], with the Entrez Gene ID as the primary identifier. An EASE score <0.05 was used to define over-represented categories [16].

### Validation of GeneChip results

Ten additional independently reared C57BL/6J male mice (Jackson Labs, Bar Harbor, ME), 3 to 5 wk of age, were utilized in the experiment. Mice were housed two to three per cage and maintained at 25°C with a 12:12-h light-dark cycle and *ad libitum *access to water and feed (Rodent Laboratory Chow, Ralston Purina, St. Louis, MO). All animals were handled in accordance with the protocol approved by the Purdue Animal Care and Use Committee. Mice were randomly assigned to one of two experimental treatments: vehicle control (C) or 24 hours (24 h) of clenbuterol treatment. Clenbuterol (Sigma, St. Louis, MO) was prepared as described previously. All mice were administered intraperitoneal injections of C or 24 h treatments at approximately 10 am. The clenbuterol treatment was administered at a dosage of 1 mg/kg of body weight, and an equivalent volume of the control treatment was injected. All mice were euthanized by CO_2 _asphyxiation 24 hours following the injection. The gracillus muscle was collected from each animal, immediately frozen in liquid nitrogen, and stored at -80°C pending RNA extraction by TRIzol (Invitrogen, Carlsbad, California).

Primers specific to mouse sequence for Arpc3, Hod, IGF1, vinculin, and GAPDH were used in the qPCR experiment (Table 7). All qPCR assays were standardized to starting quantity of RNA. One μg of total RNA was reverse transcribed using the iScript cDNA Synthesis Kit (Biorad, Hercules, CA), and one μl of the resulting cDNA was used for qPCR. The qPCR assay was carried out in the BioRad iCycler (Biorad, Hercules, CA) in a 25 μL final reaction volume. Quantification of PCR products was achieved using SYBR Green (Biorad, Hercules, CA) reagents following the manufacturer's recommended protocol with the following thermal cycling conditions: 95°C, 10 min (1 cycle); 95°C, 15 sec, 58°C, 15 sec, 72°C, 15 sec (35 cycles); 4°C. The PCR products were visualized on an agarose gel to ensure there was no non-specific PCR amplification. All assays were done in triplicate in a 96-well plate format. Standard controls were prepared by cloning the PCR product into a vector and making serial dilutions of known starting copy number. Control samples included on each 96-well plate were used to establish a standard curve for determining the log starting copy number (LSCN) of each experimental cDNA sample. The LSCN were analyzed by analysis of variance using mixed model procedures of JMP SAS (JMP 5.1, 2004). The model included treatment as a fixed effect and cDNA sample within treatment as a random effect. GAPDH was evaluated as a housekeeping gene and consistent expression was observed across experimental treatments. Therefore, no further normalization of the qPCR data was done.

## Authors' contributions

DMS designed and oversaw the research and assisted with drafting the manuscript. TGM assisted with sample collection and preparation, validated the microarray results, and assisted with drafting the manuscript. LMM participated in design of the experiment and planned and executed the microarray data analysis and assisted in writing the manuscript. All authors read and approved the final manuscript.

## Supplementary Material

Additional File 1 **Analysis results for all detected genes**. Results from all analyses completed for all genes are provided in the Excel (.xls) file. 'AffyID', 'Gene Symbol', and 'Entrez Gene ID' show the annotation used for analysis of gene ontology categories. 'Cluster' represents the cluster (1–10, see Figure 3) to which the gene belongs. 'Rank' is the relative order of overall *P*-value (lowest = 1, highest = 22,605) for all genes. '*P*-value' represents the effect of treatment (control, 24 h clenbuterol, 10 D clenbuterol) from analysis of variance. 'Normality' shows the result of testing the hypothesis that residuals from the analysis of variance are normally distributed (0 = fail to reject, 1 = reject the hypothesis, suggesting non-normal distribution of residuals). 'Contrast 24 h-C' and 'Contrast 10 D-C' and Contrast 24 h-10 D' are the *P*-values resulting from the contrast comparing the 24 h clenbuterol to control, 10 D clenbuterol to control, and 24 h clenbuterol to 10 D clenbuterol treatments, respectively. 'Mean C (log)', 'Mean 24 h (log)', and 'Mean 10 D (log)' show the mean normalized expression of the log transformed data for each treatment. These represent the values used for all statistical analyses. 'Mean C', 'Mean 24 h', and 'Mean 10 D' show the mean normalized expression of the raw data for each treatment. These values were used to estimate fold-change differences between treatments. 'GO Category' represents the Gene Ontology category to which the gene belonged, if the category was found to be over-represented by the EASE analysis. IS = intracellular signalling cascade, TL = translation, TC = transcription, AA = amino acid and derivative metabolism. Click here for file

## Figures and Tables

**Table 1:** Significantly differentially expressed genes (10% FDR threshold).

**AffyID**	**Gene Name**	**Rank^a^**	***P*-value^b^**	**Change 24 h^c^**	**Change 10 D^d^**	**Change 10 D/24 h^e^**
1424638_at	Cyclin-dependent kinase inhibitor 1A (P21)	1	3.62E-06	23.11	2.34	-9.86
1454971_x_at	TSC22 domain family, member 1	2	4.49E-06	-1.52	n/s	1.51
1417962_s_at	Growth hormone receptor	3	6.20E-06	-2.02	-1.16	1.74
1417109_at	Tubulointerstitial nephritis antigen-like	4	8.15E-06	2.36	1.40	-1.69
1439836_at	Ankyrin repeat and SOCS box-containing protein 15	5	0.0000111	-5.38	n/s	4.15
1417357_at	Emerin	6	0.0000145	5.56	n/s	-4.36
1416313_at	Myeloid/lymphoid or mixed-lineage leukaemia	7	0.0000179	-2.69	n/s	2.59
1416156_at	Vinculin	8	0.0000234	2.04	1.81	n/s
1434985_a_at	Eukaryotic translation initiation factor 4A1	9	0.0000273	1.94	1.14	-1.71
1417574_at	Chemokine ligand 12	10	0.0000281	2.77	n/s	-3.09
1458455_at	Actin-binding Rho activating protein	11	0.0000285	4.17	n/s	-4.49
1428804_at	Microfibrilar-associated protein 3-like	12^f^	0.0000346	n/s	2.04	2.40
1421425_a_at	Down syndrome critical region gene 1-like 1	13	0.0000361	-1.99	-1.29	1.55
1435824_at	YY1 transcription factor	14	0.0000363	1.40	1.28	-1.09
1448907_at	Thimet oligopeptidase 1	15	0.0000407	2.23	n/s	-1.94
1452707_at	RIKEN cDNA 4631423 FO2 gene	16	0.0000411	4.34	1.39	-3.12
1451372_a_at	ADP-ribosyltransferase 1	17	0.0000451	-2.11	n/s	2.09
1448279_at	Actin related protein 2/3 complex, subunit 3	18	0.0000453	2.43	1.24	-1.95
1416256_a_at	Tubulin, beta 5	19	0.0000563	2.33	1.23	-1.89
1440838_at	Unknown	20	0.0000619	-2.01	-1.18	1.70
1439616_at	Unknown	21	0.0000684	3.57	2.91	n/s
1449088_at	Fructose bisphosphatase 2	22	0.0000703	3.95	2.21	-1.78
1452843_at	Interleukin 6 signal transducer	23	0.0000733	1.61	1.33	-1.22
1460392_a_at	Enhancer of yellow 2 homolog	24	0.0000756	2.74	n/s	-2.42
1455218_at	Serine (or cysteine) preptidase inhibitor, clade A, member 1b	25	0.0000774	2.23	1.95	n/s
1422798_at	Contactin associated protein-like 2	26^f^	0.0000822	-2.84	-1.60	1.78
1431043_at	Kelch repeat and BTB (POZ) domain containing 5	27	0.0000929	2.36	n/s	-2.20
1427556_at	Myosin, light polypeptide kinase 2, skeletal muscle	28	0.000094	-2.26	n/s	2.37
1423396_at	Angiotensinogen	29	0.0000957	7.84	2.99	-2.62
1418571_at	Tumor necrosis factor receptor superfamily, member 12a	30	0.000104156	35.40	n/s	-15.73
1416505_at	Nuclear receptor subfamily 4, group A, member 1	31	0.000105021	-1.65	-1.16	1.43
1416328_a_at	ATPase, H+ transporting, V0 subunit E	32	0.00010965	1.76	1.56	n/s
1434303_at	Ortholog of human Ras association and pleckstrin homolog domains 1	33	0.000112542	2.60	1.60	-1.63
1438510_a_at	Histidyl-tRNA synthetase	34	0.000118695	1.46	n/s	-1.35
1418326_at	Solute carrier family 7 (cationic amino acid transporter, y+ system), member 5	35	0.000121684	13.95	6.33	-2.20
1428662_a_at	Homebox only domain	36	0.000122982	2.19	n/s	-1.98
1444232_at	Protein kinase, cGMP-dependent, type 1	37	0.000125102	-2.95	n/s	2.37
1416379_at	Pannexin 1	38	0.000125378	3.06	3.79	n/s
1449036_at	Ring finger protein 128	39	0.000144025	4.70	2.00	-2.34
1416431_at	Tubulin, beta 6	40	0.000146038	32.86	2.61	-12.59
1448484_at	S-adenosylmethionine decarboxylase 1	41	0.00015137	-3.31	-1.49	2.22
1447160_at	Unknown	42	0.00015393	1.56	1.20	-1.30
1453851_a_at	Growth arrest and DNA-damage-inducible 45 gamma	43	0.000154877	-4.28	n/s	4.90
1422557_s_at	Metallothionein 1	44	0.000164138	11.11	2.58	-4.30
1440884_s_at	Ribonucleotide reductase M2B 9TP53 inducible	45	0.000169965	-1.73	1.25	2.15
1418111_at	Bromodomain containing 4	46	0.000174896	-6.05	-2.96	2.04
1437401_at	Insulin-like growth factor 1	47^f^	0.000176747	2.22	n/s	-2.47
1426440_at	Dehydrogenase/reductase (SDR family) member 7	48	0.000182143	-1.79	-1.19	1.50
1424058_at	RIKEN cDNA 1190002C06 gene	49	0.000188845	2.99	1.61	-1.86
1455643_s_at	TSR 1, 20S rRNA accumulation, homolog	50^f^	0.000191388	2.13	n/s	-2.31
1451751_at	DNA-damage-inducible transcript 4-like	51	0.000210837	1.89	1.22	-1.54
1421466_at	Ankyrin repeat and SOCS box-containing protein 10	52	0.000224062	-3.91	n/s	2.80
1416831_at	Neuraminidase 1	53	0.000239547	1.65	n/s	-1.51
1451924_a_at	Endothelin 1	54	0.000244042	2.76	1.37	-2.02
1427400_at	Lady bird-like homeobox 1 homolog (Drosophila)	55	0.000244505	-1.82	-1.30	1.40
1425099_a_at	Aryl hydrocarbon receptor nuclear translocator-like	56	0.000246238	3.07	n/s	-3.13

^a^Rank of *P*-value based on analysis of variance of all expressed genes (n = 22,605)

^b^*P*-value resulting from analysis of variance. Model included treatment (control, 24 h, and 10 D clenbuterol administration) as a fixed effect. False discovery rates of 5, 10, and 20% correspond to *P*-values of 0.0000082, 0.00025, and 0.0012, respectively.

^c^Fold change in mRNA abundance for 24 h clenbuterol treatment relative to control. A positive fold change indicates increased mRNA abundance in 24 h treatment relative to control, a negative fold change indicates decreased mRNA abundance in 24 h treatment relative to control, and n/s indicates no significant difference between 24 h treatment and control (contrast *P*-value > 0.05).

^d^Fold change in mRNA abundance for 10 D clenbuterol treatment relative to control. A positive fold change indicates increased mRNA abundance in 10 D treatment relative to control, a negative fold change indicates decreased mRNA abundance in 10 D treatment relative to control, and n/s indicates no significant difference between 10 D treatment and control (contrast *P*-value > 0.05).

^e^Fold change in mRNA abundance for 24 h clenbuterol treatment relative to 10 D clenbuterol treatment. A positive fold change indicates increased mRNA abundance in 10 D treatment relative to 24 h treatment, a negative fold change indicates decreased mRNA abundance in 10 D treatment relative to 24 h treatment, and n/s indicates no significant difference between 10 D treatment and 24 h treatment (contrast *P*-value > 0.05).

^f^Residuals of four genes meeting the 10% FDR threshold showed evidence of a non-normal distribution.

**Table 2:** Selected^a ^genes of interest.

**AffyID**	**Gene Name**	**Rank^b^**	***P*-value^c^**	**Change 24 h^d^**	**Change 10 D^e^**	**Change 10 D/24 h^f^**
1437711_x_at	Ornithine decarboxylase, structural	70	0.000372006	1.68	1.32	-1.28
1452114_s_at	Insulin-like growth factor binding protein 5	83	0.000505398	-4.01	-1.52	2.63
1422648_at	Solute carrier family 7 (cationic amino acid transporter, y+ system), member 2	84	0.000505714	-1.81	n/s	2.23
1424112_at	Insulin-like growth factor 2 receptor	95	0.000653571	-1.58	-1.53	n/s
1448166_a_at	Proteasome (prosome, macropain) subunit, beta type 1	96	0.000658819	1.39	1.23	-1.13
1420971_at	Ubiquitin protein ligase E3 component n-recognin 1	169	0.001652967	n/s	1.75	2.12
1449547_at	Ankyrin repeat and SOCS box-containing protein 14	218	0.002452054	-2.64	n/s	2.08
1449940_a_at	Eukaryotic translation initiation factor 2B, subunit 4 delta	268	0.003245646	1.91	n/s	-1.65
1421027_a_at	Myocyte enhancer factor 2C	294	0.003678395	-1.43	n/s	1.58
1451422_at	Myosin XVIIIa	301	0.003765249	1.68	1.38	-1.22
1448171_at	Seven in absentia 2	342	0.004599517	4.39	2.57	-1.71
1451982_at	Mitogen activated protein kinase kinase 4	363	0.004917906	1.44	n/s	-1.23
1426850_a_at	Mitogen activated protein kinase kinase 6	393	0.00551809	-2.64	n/s	2.51
1448990_a_at	Myosin IB	405	0.005781979	-1.52	n/s	1.63
1423449_a_at	Actinin alpha 4	422	0.006130447	1.32	n/s	-1.69
1454664_a_at	Eukaryotic translation initiation factor 5	450	0.006788818	1.63	n/s	-1.79
1435787_at	Protein phosphatase 1 (formerly 2C)-like	454	0.006890512	-1.79	n/s	1.49
1451136_a_at	Eukaryotic translation initiation factor 2B, subunit 2 beta	455	0.006894746	1.46	n/s	-1.54
1419391_at	Myogenin	460	0.007033703	4.07	n/s	-3.61
1424268_at	Spermine oxidase	473	0.007450143	-2.54	n/s	2.89
1426833_at	Eukaryotic translation initiation factor 4 gamma, 3	569	0.009710166	-1.41	n/s	1.57

^a^Selected genes of interest that did not meet the 10% FDR criteria, but showed evidence of differential expression (*P *< 0.01) are presented and discussed in the text.

^b^Rank of *P*-value based on analysis of variance of all expressed genes (n = 22,605)

^c^*P*-value resulting from analysis of variance. Model included treatment (control, 24 h, and 10 D clenbuterol administration) as a fixed effect. False discovery rates of 5, 10, and 20% correspond to *P*-values of 0.0000082, 0.00025, and 0.0012, respectively.

^d^Fold change in mRNA abundance for 24 h clenbuterol treatment relative to control. A positive fold change indicates increased mRNA abundance in 24 h treatment relative to control, a negative fold change indicates decreased mRNA abundance in 24 h treatment relative to control, and n/s indicates no significant difference between 24 h treatment and control (contrast *P*-value > 0.05)

^e^Fold change in mRNA abundance for 10 D clenbuterol treatment relative to control. A positive fold change indicates increased mRNA abundance in 10 D treatment relative to control, a negative fold change indicates decreased mRNA abundance in 10 D treatment relative to control, and n/s indicates no significant difference between 10 D treatment and control (contrast *P*-value > 0.05)

^f^Fold change in mRNA abundance for 24 h clenbuterol treatment relative to 10 D clenbuterol treatment. A positive fold change indicates increased mRNA abundance in 10 D treatment relative to 24 h treatment, a negative fold change indicates decreased mRNA abundance in 10 D treatment relative to 24 h treatment, and n/s indicates no significant difference between 10 D treatment and 24 h treatment (contrast *P*-value > 0.05)

**Table 3:** Summary of results from cluster analysis.

**Cluster ID**	**Number of Genes**	**Percentage of Genes**
1	21,738	96.05
2	14	0.06
3	93	0.41
4	152	0.67
5	23	0.10
6	36	0.16
7	88	0.39
8	69	0.30
9	410	1.81
10	8	0.04

**Table 4:** Differentially expressed genes of interest (*P *< 0.01) categorized according to Gene Ontology biological process terms (level 2).

**Gene Ontology Biological Process Category^a^**	**Number of Genes**
Cellular physiological process	270
Metabolism	197
Cell communication	77
Regulation of physiological process	75
Localization	70
Morphogenesis	46
Organ development	38
Response to stimulus	38
Organismal physiological process	25
Regulation of cellular process	25
Cell differentiation	18
Death	14
Homeostasis	12
Regulation of development	7
Reproduction	7
Other categories^b^	32

^a^A total of 309 unique gene identifiers were placed in categories with a minimum of 2 members, while 242 remained unclassified. Categories containing a minimum of 7 members, and the number of genes belonging to the category are shown.

^b^Other categories include: embryonic development, regulation of enzyme activity, growth, mesoderm development, pattern specification, coagulation, extracellular structure organization and biogenesis, pathogenesis, rhythmic process.

**Table 5:** Evaluation of differentially expressed genes of interest (*P *< 0.01) to identify over-represented families of genes^a^.

**Gene Ontology Biological Process Category^b^**	**List Hits^c^**	**Population Hits^d^**	**EASE Score^e^**
Homeostasis (Cell homeostasis, Cation homeostasis, Cell ion homeostasis, Ion homeostasis)	10	63	.002
Amino acid and derivative metabolism (Amine metabolism, Amino acid metabolism)	16	196	.010
Intracellular signalling cascade	34	519	.021
Neurogenesis	17	209	.023
Transcription from Pol II promoter (Positive regulation of transcription)	18	229	.025
Translation (Translational initiation)	13	144	.025
Physiological processes	281	6030	.030
Morphogenesis	38	617	.034
Lymphocyte activation	6	42	.036
Protein-mitochondrial targeting	3	8	.046

^a^Data were evaluated using EASE software [66] and Gene Ontology biological process categories.

^b^Categories with evidence for over-representation (EASE score < 0.05) are shown, with redundant categories listed in parentheses.

^c^The number of genes from the list of differentially expressed genes within each over-represented category. A total of 331 differentially expressed genes were assigned to a category.

^d^The number of genes included on the MOE430A and B chips within each over-represented category. A total of 7,464 genes were assigned to a category.

^e^The EASE score is the upper bound of the distribution of Jackknife Fisher exact probabilities.

**Table 6:** Confirmation of differential gene expression 24 hours after clenbuterol administration by quantitative PCR.

	**Log of starting copy number**			
			
**Gene^a^**	**Control**	**Clenbuterol**	**Standard Error**	***P*-value**
Arpc3	4.70	5.12	0.10	0.0001
GAPDH	7.07	7.19	0.06	0.15
Hod	2.70	3.50	0.07	0.0001
IGF1	5.19	5.58	0.06	0.0001
Vinculin	3.34	3.89	0.07	0.0001

^a^Arpc3: actin related protein 2/3 complex subunit; GAPDH: glyceraldehyde 3-phosphate dehydrogenase; Hod: homeobox only domain; IGF1: insulin like growth factor 1

**Table 7:** Sequences of PCR primers used for quantitative PCR.

**Gene^a^**	**Forward Primer (5'-3')**	**Reverse Primer (5'-3')**
Arpc3	CACCAAGCTCATCGGTAACA	ATGTCCTGTCCGCTTCATTC
GAPDH	GAACATCATCCCTGCATCCA	CCAGTGAGCTTCCCGTTCA
Hod	AGCAGACGCAGAAATGGTTT	GTAAGCCGAGGGAAGGAAGA
IGF1	GGCATTGTGGATGAGTGTTG	GTCTTGGGCAGTTCAGTGTG
Vinculin	AGCTCGGAAATGGTCTAGCA	GAATAAGTGCCCGCTTGGTA

^a^Arpc3: actin related protein 2/3 complex subunit; GAPDH: glyceraldehyde 3-phosphate dehydrogenase; Hod: homeobox only domain; IGF1: insulin like growth factor 1
